# Mapping evidence on the effects of gender-affirming hormone therapy on the hard and soft tissues of the craniofacial complex in transgender people: a protocol for a scoping review

**DOI:** 10.1186/s13643-021-01668-8

**Published:** 2021-04-14

**Authors:** M. F. Van den Bosch, C. M. Wiepjes, M. Den Heijer, L. J. Schoonmade, R. E. G. Jonkman, R. A. Meursinge Reynders

**Affiliations:** 1 Department of Orthodontics, Academisch Centrum Tandheelkunde Amsterdam (ACTA), Gustav Mahlerlaan 3004, 1081 LA Amsterdam, The Netherlands; 2 Department of Internal Medicine, Amsterdam University Medical Center (Amsterdam UMC), Location VUMC, De Boelelaan 1117, 1081 HV Amsterdam, The Netherlands; 3 Center of Expertise on Gender Dysphoria, Amsterdam University Medical Center (Amsterdam UMC), Location VUMC, De Boelelaan 1117, 1081 HV Amsterdam, The Netherlands; 4 Medical Library, Vrije Universiteit Amsterdam, De Boelelaan 1117, 1081 HV Amsterdam, The Netherlands; 5 Department of Oral and Maxillofacial Surgery, Amsterdam University Medical Center (Amsterdam UMC), Location AMC, Meibergdreef 9, 1105 AZ Amsterdam, The Netherlands; 6 Studio di Ortodonzia, Via Matteo Bandello 15, 20123 Milan, Italy

**Keywords:** Transgender people, Gender incongruence, Gender dysphoria, Gender-affirming therapy, Hormonal therapy, Face, Craniofacial, Facial transition, Facial feminization, Facial masculinization

## Abstract

**Background:**

Gender-affirming hormone (GAH) therapy aims to support the transition of transgender people to their gender identity. GAHs can induce changes in their secondary sex characteristics such as the development of breasts in transgender females and increased muscle mass in transgender males. The face and its surrounding tissues also have an important role in gender confirmation. The aim of this scoping review is to systematically map the available evidence in order to provide an overview of the effects of GAH therapy on the hard and soft tissues of the craniofacial complex in transgender people.

**Methods/design:**

The Preferred Reporting Items for Systematic Reviews and Meta-Analyses Protocols (PRISMA) extension for Scoping Reviews was consulted for reporting this protocol. The methods were based on the Arksey and O’Malley’s framework and the Reviewer’s Manual of the Joanna Briggs Institute for conducting scoping reviews. Ten transgender people were involved in the development of the primary research question through short interviews. The eligibility criteria were defined for transgender people undergoing GAH therapy and for quantitative and qualitative outcomes on the hard and soft tissues of the craniofacial complex. Eligible sources of evidence include observational, experimental, qualitative, and mixed method studies. No exclusion criteria will be applied for the language of publication and the setting. To identify eligible sources of evidence, we will conduct searches from inception onwards in PubMed, Embase.com, the Cochrane Library, Web of Science Core Collection, Scopus, CINAHL, LIVIVO, and various grey literature sources such as Google Scholar. Two reviewers will independently select eligible studies in these information sources and will subsequently conduct data extraction. The same operators will chart, categorize, and summarize the extracted data. A narrative summary of findings will be conducted. Frequency counts of quantitative and qualitative data on items such as concepts, populations, interventions, and other characteristics of the eligible sources will be given. Where possible, these items will be mapped descriptively.

**Discussion:**

We chose the scoping review over the systematic review approach, because the research questions are broad-spectrum and the literature is expected to be widely scattered. No systematic review has previously assessed this topic. Identifying knowledge gaps in this area and summarizing and disseminating research findings are important for a wide spectrum of stakeholders, in particular, for transgender people who want to undergo additional interventions such as plastic or orthognathic surgery or orthodontics.

**Systematic review registration:**

This protocol was registered in the Open Science Framework: https://osf.io/e3qj6

**Supplementary Information:**

The online version contains supplementary material available at 10.1186/s13643-021-01668-8.

## Background

Gender-affirming hormone (GAH) therapy is an essential component of medical interventions for transgender people to support their gender transition [1]. This lifelong treatment induces changes in secondary sex characteristics such as the development of breasts in transgender females and muscle mass in transgender males [1]. The face also has a critical role in gender confirmation [2]. It is therefore important to understand what the effects of GAHs are on the hard and soft tissues of the face and its surrounding structures. This manuscript presents the protocol of our planned scoping review to systematically map the available evidence on this topic.

According to the International Classification of Diseases 11th Revision (ICD-11) [3], “Gender incongruence is characterized by a marked and persistent incongruence between an individual’s experienced gender and the assigned sex.” The umbrella term to describe these individuals is “transgender people.” The key terminology used in this article is listed in Table 1. A meta-analysis of 12 eligible studies showed that the prevalence of gender incongruence is 6.8 for transgender females and 2.6 for transgender males per 100.000 individuals [7]. This prevalence varies between countries and has increased during the last 50 years [7]. Gender incongruence often leads to a desire for gender transition through combinations of GAH treatment, surgical, or other health care interventions [3]. GAH therapy will lead to feminization in transgender females and masculinization in transgender males [8]. Transgender females are treated with anti-androgens (to suppress testosterone) and estrogens, where estradiol is the most important hormone. This hormone therapy is responsible for physical changes such as inducing breast development [9], reduction of muscle mass, and a change in fat distribution [10]. Transgender males are treated with androgens, of which testosterone is the dominant hormone. Androgens cause among other effects a deepening of the voice [11] and an increase in muscle mass [12].

**Table 1:** Definitions of terms

Gender incongruence	The International Classification of Diseases 11th Revision (ICD-11) [3] defines gender incongruence as “Gender incongruence is characterized by a marked and persistent incongruence between an individual’s experienced gender and the assigned sex. Gender variant behavior and preferences alone are not a basis for assigning the diagnoses in this group.”
Transgender people	T’Sjoen et al. [1] define transgender people as “An umbrella term to describe individuals whose gender identity differs from the sex assigned at birth based on their sexual characteristics.”
Transgender female	T’Sjoen et al. [1] define a transgender female as “A person who self-identifies as female, but whose sex was assigned male at birth.”
Transgender male	T’Sjoen et al. [1] define a transgender male as “A person whose sex was assigned female at birth (based on sexual characteristics) but self-identifies as male.”
Cisgender	T’Sjoen et al. [1] define cisgender as “A person whose identity matches the sex assigned at birth.”
Gender-affirming hormone (GAH) therapy^a^	For GAH therapy, we will adopt Wikipedia’s definition for transgender hormone therapy or cross-sex hormone therapy. Wikipedia [4] defines transgender hormone therapy, or cross-sex hormone therapy, as “a form of hormone therapy in which sex hormones and other hormonal medications are administered to transgender or gender nonconforming individuals for the purpose of more closely aligning their secondary sexual characteristics with their gender identity.”
Scoping review	Colquhoun et al. [5] define a scoping review as “A scoping review or scoping study is a form of knowledge synthesis that addresses an exploratory research question aimed at mapping key concepts, types of evidence, and gaps in research related to a defined area or field by systematically searching, selecting, and synthesizing existing knowledge.”
Craniofacial	The Merriam-Webster medical dictionary defines “craniofacial” as “relating to, or involving both the cranium and the face” [6]. In this manuscript, we will apply the following definition for “craniofacial”: “relating to, or involving the cranium or the face or both.”

^a^We will consider the following three terms as synonyms: gender-affirming hormone therapy, transgender hormone therapy, and cross-sex hormone therapy. In this manuscript, we will only use the term “gender-affirming hormone (GAH) therapy”

Besides the aforementioned desired changes of various parts of the body, also facial changes play a critical part in gender transition [2]. The effects of GAHs on the face and its surrounding structures could be important for transgender people especially for those planning to undergo additional facial feminization or masculinization procedures. In this article, we will assess the available evidence on the effects of these hormones on the hard and soft tissues of the craniofacial complex.

A wide body of primary and secondary research studies has been published on the general physical effects of GAHs [1], but our scoping searches did not identify any reviews that addressed our questions on this research topic. We therefore developed this protocol for a scoping review. The scoping review process was chosen over the systematic review approach, because our research questions cover a broad-spectrum topic, and the pertinent literature is expected to be widely scattered. To guarantee that our research questions addressed outcomes that are truly important for transgender people, we involved these stakeholders in the development of the primary question.

The aim of this scoping review is to systematically map the available evidence in order to provide an overview of the effects of GAH therapy on the hard and soft tissues of the craniofacial complex in transgender people. Based on this objective, we have formulated the following primary and secondary questions.

### Primary questions

What evidence is available on the effects of GAH therapy on the hard and soft tissues of the craniofacial complex in transgender people? When assessing this evidence, we will also record the adverse effects of this intervention on these tissues and other parts of the body.

### Secondary question

What evidence is available on the consequences of changes of the hard and soft tissues of the craniofacial complex as a result of GAH therapy in transgender people?

## Methods

### Reporting and conducting of the scoping review

**Fig. 1:**
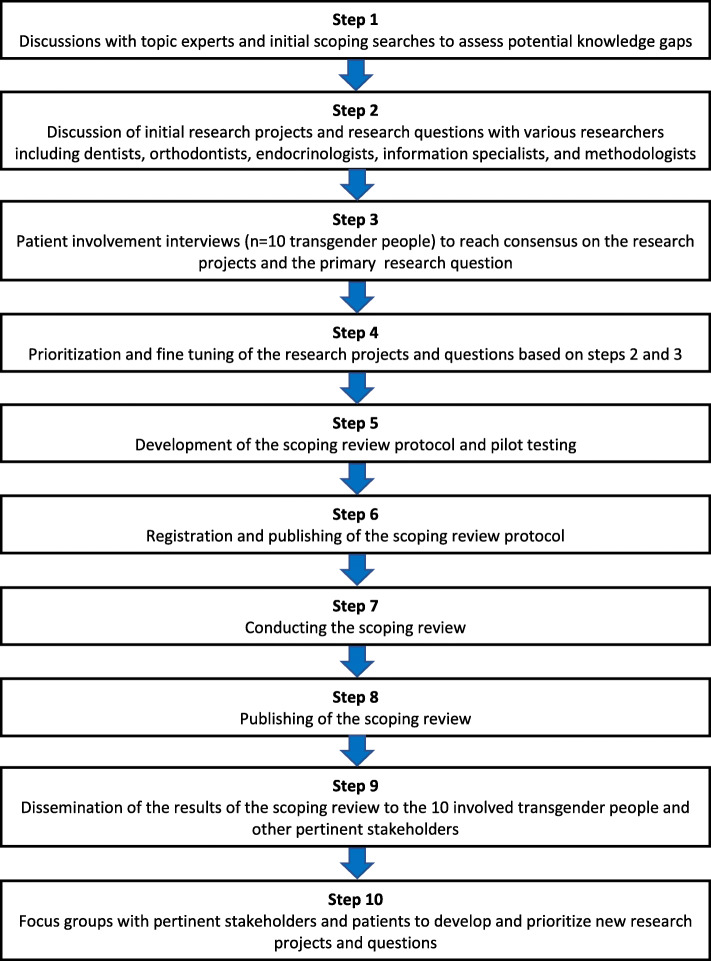
Flow diagram of the current and future research projects

This protocol is reported according to the guidelines of the Preferred Reporting Items for Systematic Reviews and Meta-Analyses Protocols (PRISMA-P) statement [13, 14] and the PRISMA extension for Scoping Reviews (PRISMA-ScR) [15] (Additional file 1). For the methods of this review, we also consulted the framework of Arksey and O’Malley’s [16] and the Reviewer’s Manual of the Joanna Briggs Institute (JBI) [17] for conducting scoping reviews. We registered our protocol a priori in the registries of the Open Science Framework (registration link: https://osf.io/e3qj6) [18]. Our planned and future research projects are reported in a flow diagram (Fig. 1).

### Patient involvement

The primary research question of this scoping review has been “co-produced” with the pertinent subjects of this research project [19]. Public and patient involvement (PPI) can improve the relevance of a research study by focusing on questions that are important to patients and to prioritize these questions [19]. We adopted the short version of the Guidance for Reporting Involvement of Patients and the Public (GRIPP2-SF) which has been developed for reporting involvement of these stakeholders in any study [19]. Our sample was selected from transgender people that consecutively visited the transgender clinic in the department of endocrinology at the Amsterdam University Medical Center, location Vrije Universiteit Medical Center. From this population, we selected the first ten subjects (7 trans women and 3 trans men) that accepted our invitation to participate in a short interview on our planned research projects and our primary research question for this scoping review. Consensus with all stakeholders was reached on our final primary research question. We will disseminate the findings of this review to all 10 participants via email. They will be given the possibility to participate in a focus group to discuss these findings and to develop future research steps (Fig. 1). Our methods for patient involvement in this research project are reported in further detail in Additional file 2A.

### Eligibility criteria

We will define the eligibility criteria for participants, interventions, outcomes, time points, settings, sources of evidence [20, 21], language, publication status, and publication dates. The inclusion and exclusion criteria for each of these items are defined in Table 2.

**Table 2:** Inclusion and exclusion criteria

Item	Inclusion criteria	Exclusion criteria
Participants	• Transgender people of any age, demographics, ethnic or socioeconomic status, and with a good medical and dental health status. • Both self-identified transgender people and those diagnosed by health professionals will be eligible. • Any term used to define transgender people will be eligible under the condition that these terms were defined by the authors and were covered by the definition for gender incongruence [3] or the umbrella definition of transgender people [1] listed in Table 1. Examples of such terms include transgender, transgender persons, trans persons, trans people, trans men, trans women, transgendered persons, transsexual persons, persons with gender dysphoria, persons with gender identity disorder, female-to-male persons, male-to-female persons, and persons with gender incongruence.	• Transgender people with congenital anomalies such as cleft lip and palate • Transgender people that underwent previous or concomitant craniofacial (e.g., plastic orthognathic) surgery
Interventions	• GAHs of any type, dosage, and administered through any type of route. • Interventions with GAHs refer to the administration of hormones such as testosterone, androgens, estrogens, estradiol, and antiandrogens. • Eligible synonyms of GAH therapy include cross-sex hormone therapy, hormone-replacement therapy, gender-confirming hormone therapy, and transgender hormone therapy. • Administration of GAHs for at least 3 months.	• GAH therapy combined with other craniofacial interventions, e.g., plastic or orthognathic surgery • GAH administration that was not supervised by health professionals
Outcomes	• Any type of quantitative or qualitative outcome of the hard or soft tissues of the craniofacial complex. We will apply the following definition for “craniofacial”: “relating to or involving the cranium or the face or both” (Table 1). • Any type of qualitative or quantitative consequences of changes in the craniofacial complex as a result of GAH therapy in transgender people.	• Outcomes associated with hair growth and dermatologic conditions such as acne
Time point	• Outcomes measured after at least 3 months of administration of GAHs.	
Setting	• Any type of setting, e.g., university or private practice setting.	
Sources of evidence	• Observational studies, i.e., exploratory, descriptive, and analytical studies. • Experimental studies, i.e., randomized trials. • Qualitative studies. • Mixed method studies.	• Systematic reviews, guidelines, editorials, viewpoints, expert opinions, commentaries, and animal studies
Language	• Any language.	
Publication status	• Only peer-reviewed manuscripts will be eligible.	
Publication dates	• Published from inception onwards.	

### Information sources

To identify eligible sources of evidence, we will conduct searches for manuscripts from inception onwards in PubMed, Embase.com, the Cochrane Library, Web of Science Core Collection, Scopus, CINAHL, and LIVIVO. The grey literature will be searched in Google Scholar, Open Grey, ClinicalTrials.gov, World Health Organization International Clinical Trials Registry Platform (WHO ICTRP), and worldwidescience.org. To find additional eligible publications, manual searches will be conducted for relevant references in included studies and in guidelines and reviews. We will contact pertinent individuals and organizations to obtain information on unpublished or ongoing studies.

### Search

Our search strategy was built around the P, I, and O elements of our PIO (Participants, Interventions, Outcomes) question. Synonyms and other relevant terms for these elements were searched in thesauri and in the primary and secondary literature on our research topic. No filters were installed. An information specialist (LS) in the health sciences at the medical library of the Vrije Universiteit Amsterdam helped in the development of customized search strategies for each information source. These strategies were subsequently peer-reviewed by a second information specialist [22, 23]. Two reviewers (MVDB and RMR) then pilot tested these strategies. A draft search strategy for MEDLINE is presented in Additional file 2B.

### Selection of sources of evidence

The selection of the sources of evidence will be based on our eligibility criteria and will be conducted independently by two reviewers (MVDB and CW). The first operator is a craniofacial and the second an endocrinology topic expert. This process will be done in 2 stages. The first stage will consist of a title and abstract screening to identify eligible publications. Rayyan, a free web and mobile app, will be used to expedite this initial screening [24]. In the second stage, the retrieved publications will be read completely, and a final selection will be made. EndNote will be used as the reference management software program [25]. Potential disagreements between the two reviewers during these selection procedures will be resolved through rereading of the pertinent manuscripts and discussions. Persistent disagreements will be resolved through independent validation by a third reviewer (RMR). To improve the transparency of our selection procedures, we will report in an additional file all references of excluded publications that could require an additional explanation for their exclusion. All steps of the selection of publications will be presented in a PRISMA flow diagram [26].

### Data charting process and data items

For the development of our data extraction forms, we consulted the checklists of the Enhancing the Quality and Transparency Of health Research Network (EQUATOR Network) [27] and the data extraction template and guidance for scoping reviews of the Joanna Briggs Institute [17]. Our pilot-tested data extraction forms with a description of each data item can be found in Additional file 2C. Our data charting process will consist of 3 steps for each eligible paper: (1) completing the data extraction forms, (2) assessing the research design of each eligible article and selecting the pertinent checklist of the EQUATOR Network [27] for this research design, and (3) completing all items of the selected EQUATOR Network checklist. These procedures will be conducted independently by the same 2 operators (MVDB and CM) that selected the sources of evidence. Disagreements between these 2 operators will be resolved through rereading the pertinent papers and discussions. A third operator (RMR) will be consulted to adjudicate persisting disagreements. This operator, a methodologist (RMR), will double-check the data extraction procedures in 25% of the eligible manuscripts. This selection of papers will be established using random numbers created by random number generator software [28].

### Quality assessment and risk of bias

Quality appraisals and formal risk of bias assessments are optional steps in scoping reviews, but are typically not conducted in such reviews [15, 16]. However, if we decide to undertake such assessments, we will describe which methods will be implemented and how these assessments will be used, e.g., in the synthesis of data if appropriate. The rationale and the consequences for this decision and the reasons for choosing the pertinent assessment tools will be given.

### Synthesis of results

We will present our evidence in a narrative format and describe how the results of each eligible article relate to our research objectives [17]. Frequency counts of quantitative and qualitative data on items such as concepts, populations, interventions, and other characteristics of the eligible sources will be reported. Where possible, we will descriptively (not analytically) map these items. We do not plan to undertake quantitative syntheses or interpretive qualitative analyses. We will consider the relevance of the publication with regard to the time of the publication, its source, and publication status. We will assess similarities or discrepancies between our PIO questions and those formulated in the included articles. Tables will be created to report the characteristics of each eligible article and the results. Extracted data items reported in Additional file 2C will be used to categorize the domains and format these tables. Tables will also explain how each article was reported according to the pertinent reporting guideline of the EQUATOR Network [27]. Tables that represent the quality and risk of bias will be given if such analyses are possible. We will also create a table that lists the identified knowledge gaps and implications for future research studies. Our findings will be disseminated to the pertinent stakeholders.

## Discussion

The proposed scoping review will systematically map the existing evidence of the effects of GAH therapy on the hard and soft tissues of the craniofacial complex in transgender people. We will assess and synthesize the literature on this research topic, identify the research gaps, consider the clinical implications, and make recommendations for future research. Any amendments made to this protocol when conducting the scoping review will be reported in the final manuscript and in the Open Science Framework. We will present the type and timing of these changes as well as the rationale and the potential consequences of these modifications.

### Strengths and limitations

The strengths of this scoping review include (1) patient involvement with the development of our primary research question; (2) the broad spectrum of information sources; (3) a research team consisting of multidisciplinary topic experts, information scientists, and methodologists; (4) pilot-tested research methods; and (5) peer-reviewed search strategies. Scoping reviews have some limitations compared with systematic reviews, i.e., registration of the review protocol is not possible in PROSPERO [29], no mandatory risk of bias assessment or critical appraisal, and no quantitative synthesis [30]. We will address the first two limitations by registering our protocol in Open Science Framework [18] and by conducting risk of bias and quality assessments when possible.

### Importance and beneficiaries

Conducting of a scoping review is important for two key reasons: (1) to identify the need to conduct research in a field when no or few primary studies will be identified and (2) as a precursor for systematic reviews when multiple studies will be identified. Whether GAHs have an effect or not on the hard and soft tissues of the craniofacial complex are both important outcomes for multiple stakeholders including transgender people, clinicians, researchers, and policymakers. Transgender people that are interested in undergoing additional medical interventions such as plastic or orthognathic surgery or orthodontics might particularly benefit from the outcomes of this review. We will disseminate our findings to all stakeholders and will discuss these outcomes in focus groups with transgender people to identify patient-important outcomes and prioritize new research questions for future studies.

## Supplementary Information

**Additional file 1:.** Checklist for the Preferred Reporting Items for Systematic review and Meta-Analysis Protocols (PRISMA-P) 2015 statement.

**Additional file 2: A**. Methods for patient involvement. **B**. Search strategy in PubMed. **C**. Data collection forms.

## Data Availability

All data generated or analyzed for this research study are reported in this manuscript.

## Abbreviations

EQUATOR Network: Enhancing the Quality and Transparency Of health Research Network
JBI: Joanna Briggs Institute
PRISMA: Preferred Reporting Items for Systematic Reviews and Meta-Analyses
PRISMA-P: Preferred Reporting Items for Systematic review and Meta-Analysis-Protocols
PRISMA ScR: Preferred Reporting Items for Systematic Reviews and Meta-Analyses extension for Scoping Reviews
WHO ICTRP: World Health Organization International Clinical Trials Registry Platform
