# Supplementary material for: Mapping evidence on the effects of gender-affirming hormone therapy on the hard and soft tissues of the craniofacial complex in transgender people: a protocol for a scoping review
Source: Syst Rev. 2021 Apr 14;10:109. doi: 10.1186/s13643-021-01668-8 (PMC8048340; doi:10.1186/s13643-021-01668-8)
Supplement: Supplementary file 2 — Additional file 2: A. Methods for patient involvement. B. Search strategy in PubMed. C. Data collection forms. [file 13643_2021_1668_MOESM2_ESM.docx]

**Additional file 2A. Methods for patient involvement**

The following steps according to the GRIPP2-SF guidance [19] were undertaken for patient involvement in this research study. This :

**Step 1: Selection of subjects**

We invited transgender people that consecutively visited the Center of Expertise on Gender Dysphoria, Amsterdam University Medical Center (Amsterdam UMC), Location VUMC to participate in a short interview on our planned research projects and questions. The first ten subjects (7 trans women and 3 trans men) that accepted our invitation to participate in this interview were selected. It was explained to these subjects that the interview was conducted by telephone.

**Step 2: Telephone interviews with subjects**

The 10 subjects that expressed their willingness to participate in this interview were contacted by telephone by one of the reviewers (MVDB). An introduction on our research study was presented by this researchers followed by a discussion and a decision on the primary research question for this scoping review. The following points were addressed during this conversation and in this order:

**Introduction**

- General background on the effects of the administration of GAHs.
- Background on the potential effects of the administration of GAHs on the craniofacial complex.
- Background on conducting systematic reviews and why such reviews are important.
- The aim of our study formulated as potential research questions and outcomes:

Primary questions: What evidence is available on the effects of GAH therapy on the hard and soft tissues of the craniofacial complex in transgender people? When assessing this evidence we will also record the adverse effects of this intervention on these tissues and other parts of the body.

Secondary questions: What evidence is available on the combined effects of GAH therapy and other facial feminization and masculinization procedures of the craniofacial complex in transgender people?

Potential outcomes: Potential consequences for the facial esthetics of the cheeks and jaws were explained.

- Why our research questions are important and for who.
- List who will benefits: the subjects of this research study, clinicians (endocrinologists, plastic surgeons, orthognathic surgeons, dermatologists, orthodontists etc.), researchers, guideline developers, and research funders.
- The dissemination of our findings of the review to the surveyees and other pertinent stakeholders.
- The involvement of the subjects in the development of future research studies on the effects of GAHs.

**Discussion and decision on the research questions**

- Discussion with the subjects. The discussion predominantly focussed on patient-important outcomes associated with the administration of GAHs on the craniofacial complex and our planned primary research question was repeated again.
- Decisions on the primary research question by the invited subjects were made. All participants were in agreement that our research topic and our primary research question were important. They were all interested in the final outcomes of our research study.

**Step 3: Reporting on the PPI in our research study**

We adopted the checklist of the GRIPP2 short form to report the pertinent items on patient involvement in research (Table GRIPP2 short form)[19]. Page numbers are given for each item.

**Table GRIPP2 short form** [19]

| **Section and topic** | **Item** | **Reported on page number** |
| --- | --- | --- |
| **1: Aim** | Report the aim of PPI* in the study | Pages 7 and 8 |
| **2: Methods** | Provide a clear description of the methods used for PPI in the study | Pages 7 and 8 |
| **3: Study results** | Outcomes—Report the results of PPI in the study, including both positive and negative outcomes | Pages 7 and 8 |
| **4: Discussion and conclusions** | Outcomes—Comment on the extent to which PPI influenced the study overall. Describe positive and negative effects | Pages 7 and 8 |
| **5: Reflections/critical perspective** | Comment critically on the study, reflecting on the things that went well and those that did not, so others can learn from this experience | Pages 7 and 8 |

*PPI: Patient and Public Involvement

**Additional file 2B. Search strategy in PubMed**

**Table. Search strategy in PubMed**

| **Search** | **Query** |
| --- | --- |
| #4 | Search: #1 AND # 2 And #3 |
| #1  Participants | "Sexual and Gender Disorders"[Mesh] OR "Gender Dysphoria"[Mesh] OR "Health Services for Transgender Persons"[Mesh] OR "Transgender Persons"[Mesh] OR "Transsexualism"[Mesh] OR "Sex Reassignment Procedures"[Mesh] OR “gender disorder*”[tiab] OR “gender identity disorder*”[tiab] OR “gender dysphor*”[tiab] OR “sexual dysphor*”[tiab] OR two-spirit*[tiab] OR transgender*[tiab] OR transsex*[tiab] OR transex*[tiab] OR trans-sex*[tiab] OR trans-gender*[tiab] OR travesti*[tiab] OR transvesti*[tiab] OR “sex reassign*”[tiab] OR “sexual reassign*”[tiab] OR “sex chang*”[tiab] OR “gender reassign*”[tiab] OR “gender chang*”[tiab] OR “gender confirm*”[tiab] OR transman[tiab] OR transmen[tiab] OR “trans man”[tiab] OR “trans men”[tiab] OR “trans person*”[tiab] OR “trans people”[tiab] OR “trans individual*”[tiab] OR transwom*[tiab] OR “trans wom*”[tiab] OR “trans ident*”[tiab] OR “trans youth”[tiab] OR “male-to-female”[tiab] OR “female-to-male”[tiab] OR “non-binary”[tiab] OR “genderfluid*”[tiab] OR “genderqueer*”[tiab] |
| #2  Interventions | "Gonadal Steroid Hormones"[Mesh] OR "Estrogens" [Pharmacological Action] OR "Androgens"[Mesh] OR "Androgen Antagonists"[Mesh] OR "sex steroid*"[tiab] OR “hormon*”[tiab] OR "estradiol"[tiab] OR "estrogen*"[tiab] OR "oestrogen*"[tiab] OR "oestradiol"[tiab] OR "progesteron*"[tiab] OR "progestagen*"[tiab] OR "testosteron*"[tiab] OR "anti-androgen*"[tiab] OR "antiandrogen*"[tiab] OR "androgen*"[tiab] |
| #3  Outcomes | "Skull"[Mesh] OR "Maxillofacial Development"[Mesh] OR "Face"[Mesh] OR “cranio-facial”[tiab] OR “craniofacial”[tiab] OR “face”[tiab] OR “facial”[tiab] OR “maxillofacial“[tiab] OR “maxillo-facial”[tiab] OR “hard tissue*”[tiab] OR “jaw”[tiab] OR “jaws”[tiab] OR “mandible*”[tiab] OR “mandibula*”[tiab] OR “chin”[tiab] OR “mentum”[tiab] OR “condylar process*”[tiab] OR “processus condylaris”[tiab] OR “maxilla*”[tiab] OR “palate*”[tiab] OR “palatine”[tiab] OR “palatum”[tiab] OR “nasal bone*”[tiab] OR “os nasale”[tiab] OR “nasal concha*”[tiab] OR “conchae nasal*”[tiab] OR “nasal septum”[tiab] OR “nasal septa”[tiab] OR “orbit*”[tiab] OR “zygoma*”[tiab] OR “cheek bone*”[tiab] OR “frontal bone*”[tiab] OR “os frontal*”[tiab] OR “soft tissue*”[tiab] OR “mouth”[tiab] OR “lip”[tiab] OR “lips”[tiab] OR “lowerlip”[tiab] OR “upperlip”[tiab] OR “vermillion border”[tiab] OR “vermilion border”[tiab] OR “vermilion show”[tiab] OR “vermillion show”[tiab] OR “gonion”[tiab] OR “parotid region*”[tiab] OR “nose*”[tiab] OR “subnasale”[tiab] OR “nasolabial”[tiab] OR “cheek*”[tiab] OR “forehead*”[tiab] OR “frontal bossing”[tiab] OR “supraorbital bossing”[tiab] OR “eyebrow*”[tiab] OR “brow”[tiab] OR “brows”[tiab] OR “hairline*”[tiab] OR “tooth”[tiab] OR “teeth”[tiab] OR “molar*”[tiab] |

**Additional file 2C. Data collection forms**

We conducted pilot tests on six articles to fine-tune the data extraction forms and our data collection methods and to calibrate reviewers. These articles cover different clinical specialties and assess interventions on our research topic.

**References of articles used for the pilot testing of the data extraction forms**

1. De Blok CJM, Klaver M, Wiepjes CM, et al. Breast Development in Transwomen After 1 Year of Cross-Sex Hormone Therapy: Results of a Prospective Multicenter Study. J Clin Endocrinol Metab. 2018;103(2):532-538. doi:10.1210/jc.2017-01927
2. Metzler P, Geiger EJ, Chang CC, Sirisoontorn I, Steinbacher DM. Assessment of three-dimensional nasolabial response to Le Fort I advancement. J Plast Reconstr Aesthet Surg. 2014;67(6):756-763. doi:10.1016/j.bjps.2014.03.023
3. Mobarak KA, Krogstad O, Espeland L, Lyberg T. Factors influencing the predictability of soft tissue profile changes following mandibular setback surgery. Angle Orthod. 2001;71(3):216-227. doi:10.1043/0003-3219(2001)071<0216:FITPOS>2.0.CO;2
4. Morris DO, Illing HM, Lee RT. A prospective evaluation of Bass, Bionator and Twin Block appliances. Part II--The soft tissues. Eur J Orthod. 1998;20(6):663-684. doi:10.1093/ejo/20.6.663
5. Saleh M, Hajeer MY, Al-Jundi A. Short-term soft- and hard-tissue changes following Class III treatment using a removable mandibular retractor: a randomized controlled trial. Orthod Craniofac Res. 2013;16(2):75-86. doi:10.1111/ocr.12007
6. Van Caenegem E, Wierckx K, Taes Y, et al. Body composition, bone turnover, and bone mass in trans men during testosterone treatment: 1-year follow-up data from a prospective case-controlled study (ENIGI). Eur J Endocrinol. 2015;172(2):163-171. doi:10.1530/EJE-14-0586

**Data collection form 1. Article information***

| **Entry** | **Description** |
| --- | --- |
| Reference | Give reference of article |
| Information source | Source of article, e.g., retrieved in PubMed, through handsearching of references etc. |
| Language | Report language of article. |
| Country | Report the country where the study was conducted. |
| Country institute | Report the country of the first institute listed. |
| Objectives | Copy and paste the objectives of the article. |
| Funding | Report the funding and other support (e.g., providing the gender-affirming hormones) as well as the role of funders |

*For each entry give the page number and column, e.g., page 12 column 2

**Data collection form 2. Study design and registration***

| **Entry** | **Description** |
| --- | --- |
| Study design | Describe the study design, e.g., randomized controlled trial, cohort study etc. |
| Ethical review board | Approved by ethical review board/Not reported. |
| Trial register | Registration number trial/Not registered/Not reported. |
| Protocol register | Protocol registration number/Not registered/Not reported. |
| Risk of bias | Describe design and registration related biases that could have an effect on the outcomes of the article. |

*For each entry give the page number and column, e.g., page 12 column 2

**Data collection form 3. Participants* ****

| **Entry** | **Description** |
| --- | --- |
| Eligibility criteria | Report the eligibility criteria for participants. |
| Recruitment | Report the recruitment methods of the participants. |
| Number | Report the total number of included participants. |
| Transwomen/transmen | Report the number of included transwomen/transmen. |
| Age | Report age of included participants. Give age in years and months with standard deviations and/or ranges. |
| Ethnicity | Report the ethnicity of included participants. |
| Socio-economic status | Report the socio-economic status of included participants. |
| Physical status | Report the physical status of included participants. Report health issues such as diabetes, smoking, pharmacological treatment etc. |
| Dental status | Report the dental status of included participants. Report dental health issues such as periodontal disease, loss of teeth etc. |
| Co-existent conditions | Report co-existent conditions of included participants. Report past interventions or co-interventions etc. |
| Additional participant characteristics | Report additional characteristics of included participants. |
| Risk of bias*** | Describe participants related biases that could have an effect on the outcomes of the article. |

* For each entry give the page number and column, e.g., page 12 column 2

** If applicable give also the number of participants per subgroup with a specific entry

*** Risk of bias judgments are assigned for participants-related conduct in the article. This assignment will exclusively focus on the assessment of conduct and we will not consider how various entries on participants were reported. We will assign risk of bias judgments for one of three levels: Low risk of bias, some concerns, or high risk of bias.

**Data collection form 4. Interventions* ****

| **Entry** | **Description** |
| --- | --- |
| Eligibility criteria | Report the eligibility criteria for interventions. |
| Gender Affirming Hormone (GAH) type | Report the type of GAH that was administered. |
| Dosage and frequency of administration of GAHs | Report the dosage and frequency of administration of GAHs. |
| Duration of administration of GAHs | Report the duration of administration of GAHs. |
| Route of administration of GAHs | Report the route of administration of GAHs. |
| Additional intervention characteristics | Report additional intervention characteristics. |
| Risk of bias*** | Describe intervention related biases that could have an effect on the outcomes of the article. |

* For each entry give the page number and column, e.g., page 12 column 2

** If applicable give also the number of participants per subgroup with a specific entry

*** Risk of bias judgments are assigned for intervention-related conduct in the article. This assignment will exclusively focus on the assessment of conduct and we will not consider how various entries on interventions were reported. We will assign risk of bias judgments for one of three levels: Low risk of bias, some concerns, or high risk of bias.

**Data collection form 5. Outcomes and time points* ****

| **Entry** | **Description** |
| --- | --- |
| Eligibility criteria | Report the eligibility criteria for outcomes. |
| Outcomes | Report the type of outcome that was measured. Outcomes could refer to changes of cephalometric and soft tissue facial landmarks etc. |
| Composite outcomes | Report potential composite outcomes. |
| Surrogate outcomes | Report potential surrogate outcomes. |
| Outcome measures | Report outcomes measures used such as mean difference, risk ratios, visual analogue scale scores etc. |
| Outcome assessors | Report the number of outcome assessors. |
| Calibration outcome assessors | Report whether outcome assessors were calibrated and how. |
| Methods for measuring outcomes | Report whether outcome assessors measured outcomes independently and whether and how they were blinded. |
| Time point(s) | Report the time point(s) for measuring outcomes. |
| Additional outcomes | Report additional outcomes. |
| Risk of bias*** | Describe outcome and time point related biases that could have an effect on the outcomes of the article. |

* For each entry give the page number and column, e.g., page 12 column 2

** If applicable give also the number of participants per subgroup with a specific entry

*** Risk of bias judgments are assigned for outcome and time points-related conduct in the article. This assignment will exclusively focus on the assessment of conduct and we will not consider how various entries on outcomes and time points were reported. We will assign risk of bias judgments for one of three levels: Low risk of bias, some concerns, or high risk of bias.

**Data collection form 6. Adverse effects and time points* ****

| **Entry** | **Description** |
| --- | --- |
| Adverse effects | Report the type of adverse effects that were measured. |
| Adverse effect measures | Report adverse effect measures used such as mean difference, risk ratios, visual analogue scale scores etc. |
| Adverse effect assessors | Report the number of outcome assessors of adverse effects. |
| Calibration adverse effect assessors | Report whether outcome assessors were calibrated and how for measuring adverse effects. |
| Methods for measuring adverse effects | Report whether outcome assessors measured adverse effects independently and whether and how they were blinded. |
| Time point(s) for measuring adverse effects | Report the time point(s) for measuring adverse effects. |
| Risk of bias | Describe adverse effects and time point related biases that could have an effect on the outcomes of the article. |

* For each entry give the page number and column, e.g., page 12 column 2

** If applicable give also the number of participants per subgroup with a specific entry

**Data collection form 7. Setting* ****

| **Entry** | **Description** |
| --- | --- |
| Setting | Report the setting such as university or private institutes etc. |
| Risk of bias*** | Describe setting related biases that could have an effect on the outcomes of the article |

* For each entry give the page number and column, e.g., page 12 column 2

** If applicable give also the number of participants per subgroup with a specific entry

*** Risk of bias judgments are assigned for setting-related conduct in the article. This assignment will exclusively focus on the assessment of conduct and we will not consider how various entries on settings were reported. We will assign risk of bias judgments for one of three levels: Low risk of bias, some concerns, or high risk of bias.

**Data collection form 8. Statistics***

| **Entry** | **Description** |
| --- | --- |
| Sample size | Report how sample size was determined. |
| Statistics | Report what statistics were used in this manuscript, e.g., to compare groups. |
| Additional issues on statistics | Report additional issues on statistics in the article. |
| Risk of bias** | Describe statistics related biases that could have an effect on the outcomes of the article |

* For each entry give the page number and column, e.g., page 12 column 2

** Risk of bias judgments are assigned for statistics-related conduct in the article. This assignment will exclusively focus on the assessment of conduct and we will not consider how various entries on statistics were reported. We will assign risk of bias judgments for one of three levels: Low risk of bias, some concerns, or high risk of bias.

**Data collection form 9. Differences between the protocol and the completed study***

| **Entry** | **Description** |
| --- | --- |
| Differences between the protocol and the completed study | Report potential differences between the protocol and the completed study and whether and how this could influence the outcomes of the study. |

* For each entry give the page number and column, e.g., page 12 column 2

**Data collection form 10. Results***

| **Entry** | **Description** |
| --- | --- |
| Results | Report the results of the study. We will apply the checklists of the EQUATOR Network for each specific study design to extract the pertinent issues for the results [27]. |

* For each entry give the page number and column, e.g., page 12 column 2

**Data collection form 11. Discussion***

| **Entry** | **Description** |
| --- | --- |
| Strengths | Report the strengths of the article. |
| Limitations | Report the limitations of the article. |
| Generalizability | Report the generalizability (external validity and applicability) of the article findings. |
| Interpretation | Report whether the interpretation of the findings are consistent with the results, the balance between beneficial and adverse effects, and possible other relevant variables. |
| Additional issues | Report additional issues that have not been covered in the various data collection forms. |

* For each entry give the page number and column, e.g., page 12 column 2
